# Reducing CKD burden in Europe: first make the elephant apparent!

**DOI:** 10.1093/ckj/sfaf182

**Published:** 2025-06-12

**Authors:** Luca De Nicola, Roberto Minutolo, Giuseppe Grandaliano

**Affiliations:** Nephrology Unit-Department of Advanced Medical and Surgical Sciences, University of Campania Luigi Vanvitelli in Naples, Italy; Nephrology Unit-Department of Advanced Medical and Surgical Sciences, University of Campania Luigi Vanvitelli in Naples, Italy; Department of Translational Medicine and Surgery, Università Cattolica del Sacro Cuore, and Nephrology Unit, Department of Medical and Surgical Sciences, Fondazione Policlinico Universitario A. Gemelli, Rome, Italy

A recent paper has described chronic kidney disease (CKD) as an elephant as compared with the four noncommunicable diseases (NCDs) recognized by the World Health Organization (WHO), namely cardiovascular diseases, pulmonary diseases, diabetes mellitus and cancer [1]. We fully agree with this picture because CKD has now surpassed the four major NCDs in terms of size of patient population, mortality rate and socioeconomic costs [2]. In the last year, the major societies of nephrology worldwide have collectively asked to add CKD to the list of NCDs acknowledged by WHO [2]. Remarkably, WHO has now positively responded to this request by adopting the ‘kidney health’ resolution during the 78th World Health Assembly held in Geneva on 23 May 2025. As highlighted by the International Society of Nephrology, this is an historic step: for the first time kidney health has been formally prioritized within the WHO NCD agenda, paving the way for earlier detection, better prevention and improved access to treatment (https://www.theisn.org/blog/2025/05/23/historic-win-for-kidney-health-as-who-adopts-global-resolution/). We therefore believe that it is time for nephrologists to start working with local healthcare institutions and politicians in order to increase awareness and detection of CKD to thereby ‘make the elephant apparent’. We here address this major unmet need of contemporary nephrology.

Early detection of CKD is essential for timely implementation of nephro- and cardio-protective interventions aimed at lowering the high cardiorenal risk. The most recent and extensive meta-analysis of 27.5 million subjects from general population and CKD cohorts has demonstrated that lower estimated glomerular filtration rate (eGFR) and more severe albuminuria herald a higher risk for as many as 10 different adverse events [3]: kidney failure onset, cardiovascular and all-cause mortality, acute kidney failure, all-cause hospitalizations, coronary artery disease, stroke, heart failure, atrial fibrillation and peripheral artery disease. Whether this holds true also in elderly patients with a slightly lower eGFR (59–45 mL/min/1.73 m^2^) as an expression of kidney senescence remains a matter of debate [4]. Notably, these complications raise the costs of CKD even before the patient reaches the most expensive phase of kidney replacement therapy, and also outweigh the financial burden of diabetes, cancer and cardiovascular events [2]. Of note, CKD also limits the implementation of extra-nephrological life-saving interventions for cardiological, infectious and oncological diseases. The whole scenario further worsens when considering the significant growth of CKD population expected in the future [5].

Consequently, CKD management has three key objectives: enabling life-saving therapies, slowing the progression of kidney damage to the deadly and expensive replacement therapy, and limiting the worsening of cardiovascular damage invariably associated with progressive CKD. Nephrologists could play a crucial role if present in sufficient number, because their involvement can significantly improve CKD prognosis, potentially leading to disease remission once the novel nephroprotective agents are added on top of traditional treatment [6, 7]. In addition, proper involvement of nephrologists in the care of CKD may be helpful to reduce the high discontinuation rates of recommended nephroprotective agents by limiting adverse renal events [8]. Alternatively, in the absence of an adequate number of nephrologists, general practitioners (GPs) can take care of most CKD patients, as it already occurs in UK where patients are often seen only by GPs up to stage 4. Indeed, it is estimated that, worldwide, the size of CKD population will grow in parallel with the shrinkage of the nephrology task force.

Regardless of the clinical setting, either tertiary nephrology care or primary care, we are facing a ‘clinical paradox’ because despite the fact that multiple therapies proven to slow CKD progression are available today, their implementation is restricted to only a minority of eligible patients due to poor CKD detection. To date, in fact, only 10%–20% of CKD patients are aware of their condition and are therefore potentially treated [6, 7, 9, 10]. The three main reasons for this phenomenon are: (i) CKD is asymptomatic until its advanced stages, meaning that most patients are unaware of their kidney disease, (ii) parameters for diagnosing kidney disease are often not included in the general health assessments, and (iii) even when kidney function is estimated, most patients with stage 3 CKD (about 80%) remain undiagnosed due to limited awareness among GPs and non-nephrologist specialists. A KDIGO panel highlighted this issue and recommended specific screening programs worldwide to identify and treat CKD patients in order to lessen CKD progression and the associated human, social and economic burden [6, 7].

Accordingly, the call for action launched by international societies to reduce the CKD burden should be first centred on a proactive screening of CKD, that is, identification of patients preliminary to timely implementation of innovative therapies. This latter task has been accomplished so far by spreading info on CKD through clinical meetings. However, ‘scientific’ communication *per se* seems not be sufficient as awareness and diagnosis of CKD still remain unacceptably low, in part because dissemination of knowledge does not primarily involve GPs. In this perspective, the ERA Strong Kidney taskforce, in collaboration with the European Kidney Health Alliance and the European Kidney Patients Federation, recently launched the ABCDE campaign in order to advocate for individuals and their GPs adopting an easy five-step approach for detecting CKD (Albuminuria, Blood pressure, Cholesterol, Diabetes, eGFR) [11].

Under this view, the direct involvement of policymakers can be extremely useful. In the last year, the Italian Society of Nephrology (SIN) has started a dialogue with politicians in face-to-face meetings, with the final objective of introducing a bill to the National Congress on the proactive screening of CKD in high-risk subjects. On several official occasions, our society presented data on CKD burden and the potential advantages of early identification and treatment of disease to lawmakers, and disseminated updates on the advancement of the proposal through print and broadcast media. The Italian National Kidney Foundation, the main GP’s associations and patient advocacy groups actively supported this initiative.

**Figure 1:**
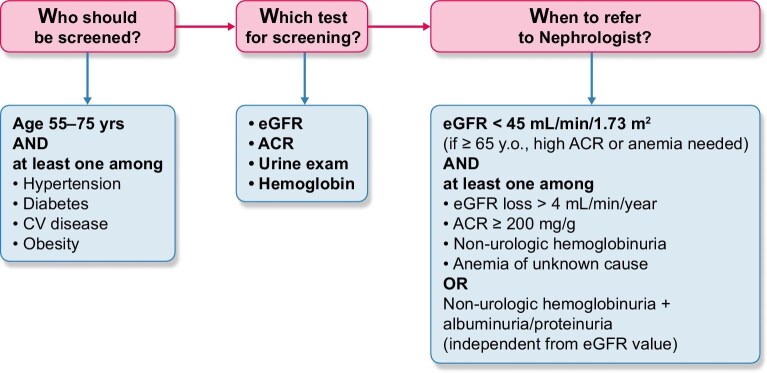
The 3W approach to identify and refer progressive CKD to nephrology care (modified from Luca De Nicola *et al*. ‘Slowing progression and preventing complications of chronic kidney disease’, ERA Neph-Manual 2023).

On 19 March 2025, the bill began the official legislative process to become law, likely by the end of this year. The bill includes a streamlined strategy to be implemented in the offices of GPs willing to participate into the national program (Fig. 1). We identified the primary care setting for screening CKD because GPs see the majority of patients at risk of CKD and can effectively collaborate with nephrologists for (i) management of early CKD stages and (ii) referral to nephrology clinics of those patients with more severe or progressive disease [12]. The direct involvement of GPs may also prevent the main drawback of population-wide screenings made as part of health examination surveys or based on home testing [9, 13], where participation rate was high but only a minority of patients with CKD diagnosis visited their GP to receive proper CKD treatment. The involvement of GPs will not be limited to the screening activity; SIN and the major national GPs societies will jointly organize educational meetings to provide GPs with updated knowledge on management of mild-to-moderate CKD.

We designed a simple and sustainable screening plan that considers the already busy practice of GPs as the limited resources available for prevention programs in Italy (Fig. 1). In order to diagnose and categorize CKD, we will use low-cost tests that are easily available, that is, serum creatinine to estimate GFR, urinary albumin–creatinine ratio (ACR) to quantify albuminuria and urine dipstick testing for the presence of haemoglobinuria (after exclusion of urologic causes); we will also measure haemoglobin level because low haemoglobin, like high albuminuria, permits identification of true progressive CKD in older subjects [14]. Interestingly, GPs have expressed their preference for this pathophysiological approach over using an eGFR value below an age-specific threshold to define CKD in elderly patients. Screening will be limited to patients at high risk of CKD. To this aim, we will test only subjects aged 55–75 years, which is the most cost-effective age range for CKD screening preliminary to treatment [15], with at least one risk factor among hypertension, diabetes mellitus, heart disease or obesity, which in Italy represents 75%, 30%, 18% and 39%, respectively, of the CKD population extracted from the National Health Examination Survey [9]. The subjects found with kidney disease will repeat exams after 3–6 months to confirm diagnosis of CKD. The final step will be the referral to the nearest nephrology unit of patients identified to have progressive or severe CKD, to diagnose the underlying primary kidney disease and optimize management. A simple form, included in the software used by GPs in their clinical practice, will guide them throughout the screening procedure up to the indications for referral to the nephrology clinic.

The proposed model may reasonably be a starting point for discussion aimed at building a European vision on CKD-screening programs, under the umbrella of ERA, which is global and local at the same time. Indeed, the aim to inprove CKD detection is global and follows the recommendations of KDIGO 2024 guidelines, whereas the methodology for testing and referral must be local in order to fit with the different healthcare systems. In those countries where GPs play a major or exclusive role in managing most CKD patients, a potential alternative is to generate an algorithm by artificial intelligence that may be implemented in the software that GPs use to manage the electronic medical records; the algorithm will be aimed at distinguishing individuals with progressive or severe disease that need nephrology consultation from those with stable CKD that can be managed by GPs only. In the latter case, GPs can also be helped to guide therapy by alerts on abnormal lab values, and telemedicine consultation with a nephrologist as well. Resources to develop and implement dedicated algorithms and telemedicine may derive from European health initiatives such as the EU4Health programme and Digital Europe.

Improving kidney health across Europe is the main mission of ERA which to this aim should dialogue with European policymakers to place kidney diseases in the list of top priorities of the European Commission for Public Health. This is the time to share in Europe health initiatives, involving politicians, in order to contribute to ‘Make Europe Great Again’… at least from the nephrology point of view.
